# Determining resident microbial community members and their correlations with geochemistry in a serpentinizing spring

**DOI:** 10.3389/fmicb.2023.1182497

**Published:** 2023-06-15

**Authors:** Leah R. Trutschel, Brittany R. Kruger, Joshua D. Sackett, Grayson L. Chadwick, Annette R. Rowe

**Affiliations:** ^1^Department of Biological Sciences, University of Cincinnati, Cincinnati, OH, United States; ^2^Division of Hydrologic Sciences, Desert Research Institute, Las Vegas, Las Vegas, NV, United States; ^3^Department of Molecular and Cell Biology, University of California, Berkeley, Berkeley, CA, United States

**Keywords:** serpentinization, Ney Springs, Illumina 16S rRNA, metagenome, sulfur oxidation intermediates

## Abstract

Terrestrial serpentinizing systems allow us insight into the realm of alkaliphilic microbial communities driven by geology in a way that is frequently more accessible than their deep subsurface or marine counterparts. However, these systems are also marked by geochemical and microbial community variation due to the interactions of serpentinized fluids with host geology and the surface environment. To separate the transient from the endemic microbes in a hyperalkaline environment, we assessed the Ney Springs terrestrial serpentinizing system microbial community and geochemistry at six time points over the span of a year. Using 16S rRNA gene surveys we observed 93 amplicon sequence variants (ASVs) that were found at every sampling event. This is compared to ~17,000 transient ASVs that were detected only once across the six sampling events. Of the resident community members, 16 of these ASVs were regularly greater than 1% of the community during every sampling period. Additionally, many of these core taxa experienced statistically significant changes in relative abundance with time. Variation in the abundance of some core populations correlated with geochemical variation. For example, members of the *Tindallia* group, showed a positive correlation with variation in levels of ammonia at the spring. Investigating the metagenome assembled genomes of these microbes revealed evidence of the potential for ammonia generation via Stickland reactions within *Tindallia*. This observation offers new insight into the origin of high ammonia concentrations (>70 mg/L) seen at this site. Similarly, the abundance of putative sulfur-oxidizing microbes like *Thiomicrospira*, *Halomonas*, and a *Rhodobacteraceae* species could be linked to changes observed in sulfur-oxidation intermediates like tetrathionate and thiosulfate. While these data supports the influence of core microbial community members on a hyperalkaline spring’s geochemistry, there is also evidence that subsurface processes affect geochemistry and may impact community dynamics as well. Though the physiology and ecology of these astrobiologically relevant ecosystems are still being uncovered, this work helps identify a stable microbial community that impacts spring geochemistry in ways not previously observed in serpentinizing ecosystems.

## Introduction

Serpentinization is a globally relevant subsurface process caused by the hydration of iron and magnesium rich minerals within the Earth’s crust which subsequently releases hydrogen gas (Mccollom and Seewald, 2013). The hydrogen produced, in addition to other reduced compounds generated, can serve as the energetic basis for microbial food webs. However, the high pH fluids overall have a profound effect on habitability. The degree of serpentinized fluid input can greatly alter microbial community composition, with pH in particular cited as a significant driver in systems that experience a range of pH values (Rempfert et al., 2017; Twing et al., 2017; Fones et al., 2021). The interaction of high pH serpentinized fluids with local geology and other water sources can also result in variation at the microbial community level, even within the same system (Morrill et al., 2013; Rempfert et al., 2017; Ortiz et al., 2018). Lastly, time scale can also have an effect on community composition, as sites undergoing active serpentinization are more impacted by high pH fluids than inactive ones (Schrenk et al., 2004; Szponar et al., 2013). Despite these known broad effects on microbial community composition, we still have limited insight into the specific geochemical drivers that explain the differences seen in the microbial communities across these systems.

Remarkable variation is seen across continental serpentinizing systems, even when comparing ones that are located within the same geologic formation (Woycheese et al., 2015; Trutschel et al., 2022). For example, Ney Springs and The Cedars are both a part of the Franciscan Subduction complex, but feature very different levels of salinity and are dominated by different microorganisms (Suzuki et al., 2017; Cook et al., 2021; Trutschel et al., 2022). Ney Springs is a terrestrial system notable for its extremely high pH (12.3–12.7) and abundance of ammonia, methane, and sulfide compared to other serpentinizing systems (Cook et al., 2021; Trutschel et al., 2022). Despite its continental location it also has marine-like levels of sodium, potassium, and boron which are likely the result of serpentinized fluids mixing with connate seawater and/or the Franciscan subduction complex marine deposit (Feth et al., 1961; Barnes et al., 1972). Ney Springs also contains incredibly high amounts of silica (>4,000 mg/L) which is likely due to the hyperalkaline fluids dissolving nearby silica-rich volcanic rocks (Feth et al., 1961; García-Ruiz et al., 2017). Ney Springs is dominated by members belonging to *Tindallia* and *Izimaplasma*, which are not typically abundant or even observed within other characterized serpentinizing systems (Trutschel et al., 2022). In comparison, The Cedars is known for its low conductivity fluids and a shallow groundwater microbial community that is dominated by the alkaliphilic and hydrogenotrophic *Serpentinomonas* (Morrill et al., 2013; Suzuki et al., 2013, 2017). Conductivity values at The Cedars are much lower compared to Ney Springs (0.8–3.0 mS/cm vs. 32–39 mS/cm, respectively), and The Cedars is limited for terminal electron acceptors such as sulfate and nitrate (Suzuki et al., 2013, 2017). The geochemical differences observed in these environments are likely explained by local variation in geology and hydrology, which in turn shape the microbial community composition and the challenges these microorganisms face.

Though surface exposed terrestrial systems are generally more easily accessed compared to their marine or purely deep subsurface counterparts, they are also subject to greater exogenous inputs and/or may be more impacted by seasonality (e.g., through precipitation, temperature, or photoperiod). Thus, they likely contain a mixture of microorganisms sustained solely by deep subsurface fluid chemistry, and microorganisms that utilize nutrient inputs and/or oxygen resulting from surface exposure. Long term geochemical and microbial community monitoring has been used to study temporal changes and the surface influence on the microbial community composition of mines and soda lake environments (Boros et al., 2017; Osburn et al., 2019). This approach allows one to determine what the endemic microbial community members of these interface environments are, how they utilize both subsurface and surface resources, and how they are impacted by temporal or seasonal changes in the environment.

In this work we assess the microbial community and aqueous geochemistry at Ney Springs over several points in a year (May 2021 – June 2022). This work identifies geochemical parameters at Ney Springs that change seasonally and those that vary temporally and are not associated with seasonality. We also present data identifying a core microbial community with an average seasonal relative abundance greater than 1%. Using metagenomics, we then investigated the potential metabolic features of this core community and how microbial metabolism may link to geochemical variation observed in this environment.

## Materials and methods

### Sample collection and analysis of aqueous geochemistry

Samples and field work were conducted at Ney Springs roughly every two months starting in May of 2021 through June of 2022 for a total of six trips. All Ney Springs fluids were collected from a 1 m × 1 m concrete cistern which captures the spring discharge (Supplementary Figure 1). A Mettler-Toledo multimeter (Columbus, OH, United States) was used to measure temperature, pH, conductivity, total dissolved solids (TDS), resistivity, and oxidation–reduction potential (ORP). Geochemical analyses conducted on site for dissolved oxygen (DO), S^2−^, Fe^2+^, tetrathionate (S_4_O_6_^2−^), and thiosulfate (S_2_O_3_^2−^) were done with a HACH (Loveland, CO, United States) portable spectrometer as described previously (Trutschel et al., 2022).

Fluid samples for ion chromatograph (IC) analysis of anions (F^−^, Cl^−^, NO_2_^−^, Br−, NO_3_^−^, PO_4_^3−^, and SO_4_^2−^) and cations (Li^+^, Na^+^, NH_4_^+^, K^+^, Mg^2+^, and Ca^2+^) were collected using autoclaved MasterFlex^®^ PharMed^®^ BPT tubing (Cole-Palmer, Vernon Hills, IL, United States) with a Geopump^™^ peristaltic pump (GeoTech, Denver, CO, United States) to pump up water from the bottom of the cistern (Supplementary Figure 1). Fluids were passed through a polypropylene in-line filter housing (Millipore; Bedford, MA, United States) containing 0.1 μm polycarbonate membrane filters (47 mm diameter, Millipore, Tullagreen, Carrigtwohill Co. Cork, IRL) and kept on ice or refrigerated (4°C) until analysis on a Dionex Aquion Ion Chromatograph (Thermo Fisher Scientific, Waltham, MA, United States). All samples were run at a 1:10 dilution with MilliQ water, or at a 1:5 dilution after the sample had been mixed with Amberlite^®^ MB20 H/OH resin beads (Sigma-Aldrich, United States, with a ratio of 80 mg of beads per 2 mL of sample) for chloride removal. This allowed for better detection of less abundant constituents such as NO_3_^−^ and NO_2_^−^. Additional samples were collected for external analysis through ACZ Laboratories (acz.com) (Steamboat Springs, CO, United States) for metals (silicon, iron, sodium, etc.) and ion species (nitrate, nitrite, phosphate, sulfate, and sulfide) using sample bottles and protocols provided by the company. Briefly, filtered, and unfiltered fluids were added to 250 mL HDPE bottles that were empty or contained 2 mL of 50% HNO_3_. Bottles were kept on ice (~4°C) then shipped within 24 h and analyzed at ACZ. Inductively Coupled Plasma Spectroscopy (ICP) according to EPA Method 200.7 was used for metal analysis. EPA methods M353.2, M350.1 and M365.1 were used for nitrate/nitrite, ammonia, and phosphorous/phosphate, respectively. Methods D516-02/-07/-11 and SM4500s2-D were used for sulfate and sulfide (total sulfides or S). Samples for stable isotope analysis of hydrogen and oxygen in water were collected in glass exetainer vials filled without headspace or bubbles and capped to prevent evaporation and exchange of samples with atmospheric water vapor. Samples were analyzed at the Center for Stable Isotope Biogeochemistry at the University of California, Berkeley using Isotope Ratio Mass Spectrometry (IRMS). Stable isotope results were reported in parts per thousand (‰), using standard delta notation (δ^2^H and δ^18^O) and are relative to VSMOW (Vienna Standard Mean Ocean Water).

### Sample collection, DNA extraction, 16S rRNA gene and metagenomic sequencing

Microbial biomass was collected using the aforementioned peristaltic pump, 0.1 μm polycarbonate membrane filters, and filter setup. Filter housings were kept on ice in the dark while pumping water during summer collection periods. Water was pumped through the filters until they clogged, which occurred over a range of 2–12 L, then filters were promptly harvested and preserved on dry ice and then later at −20°C. At minimum three filters were obtained from the Ney Springs cistern at each collection period. DNA was extracted from preserved filters alongside an unused filter from the same pack to serve as a blank control using a Qiagen DNAeasy Powersoil kit. DNA was then quantified using a Qubit fluorometer (ThermoFisher Scientific, United States). Samples were then sent to Novogene (en.novogene.com) (Beijing, CHN) for 16S rRNA NovaSeq PE250 amplicon sequencing targeting the V4 region (515F-806R) using the Earth Microbiome project primers and protocol (Thompson et al., 2017) or for paired end 150 shotgun metagenomic sequencing.

### 16S rRNA analysis

Raw sequence data was trimmed, chimera checked, and quality filtered in DADA2 (V. 1.22) (Callahan et al., 2016) for R (V. 4.1.2). Taxonomic classification was performed using a compatible Naive Bayesian classifier trained using the SILVA_nr99_V138 training set implemented for DADA2 (McLaren, 2020). Phyloseq (V. 1.38.0) was used to generate taxonomic bar charts for 16S rRNA gene data (Mcmurdie and Holmes, 2013). Contamination sequences were determined by using the blank filter controls and were removed from the dataset using the prevalence based method in Decontam, which compares the presence/absence of taxa found in contaminated control samples to that in actual samples (Davis et al., 2018). For determination of core community Amplicon Sequence Variants (ASVs), 23 samples were first pooled into six categories based on time of sampling and then assessed for ASV detection. ASVs that were found within all six sampling events were deemed resident community members, while ASVs found during only one of the six sampling events were deemed transient. Microbial community composition and seasonal taxa overlap were visualized by generating an upset plot in UpSetR (V. 1.4.0) (Conway et al., 2017). Comparisons of mean relative abundance of core community ASVs was done using a Kruskal-Wallis test from the Vegan R package (V. 2.6-2) (Oksanen et al., 2022) followed by a Dunn test adjusted with Benjamini-Hochberg correction. The MicroViz R package (Barnett et al., 2021) was used to performed a redundancy analysis (RDA) on community samples with ASVs only detected once over the six sampling periods removed from the sample pool. Changes in the relative abundance of core community ASVs were compared with changes in various geochemical species overtime by calculating the correlation coefficient in Excel. 16S rRNA gene amplicon sequences are available under the NCBI BioProject accession number PRJNA739719.

### Metagenome analysis

Metagenome sequencing data was pooled from the July 2021, January 2022, and March 2022 sampling trips as we were able to obtain sufficient biomass for metagenomics during these trips. Initial taxonomic classification of metagenome reads was performed with Kaiju (v1.7.4) (Menzel et al., 2016). Metagenomic reads were obtained and co-assembled by IDBA-ID (v1.1.3) (Peng et al., 2012), MEGAHIT (v1.2.9) (Li et al., 2015) and metaSPAdes (Nurk et al., 2017) (v 3.15.3) within the Kbase web platform (Arkin et al., 2018). The three assemblies were then binned using CONCOCT (v1.1) (Alneberg et al., 2014), MaxBin2 (V2.2.4) (Wu et al., 2016) and MetaBAT2 (v1.7) (Kang et al., 2019) for a total of nine different permutations. DAS tool (v1.1.2) (Sieber et al., 2018) was then used to merge any overlapping or redundant bins generated from CONCOCT, MaxBin2, and MetaBAT2 into one set of bins for each of the three assembly methods. These three assemblies were then classified using GTDB-tk (v1.7.0) (Parks et al., 2018) and extracted as bins. The bins were named for their number, taxonomic classification, and assembly method and were then merged as one large assembly set which was assessed in CheckM (v1.0.18) (Parks et al., 2015). A multiple sequence alignment (MSA) is generated in CheckM with HMMER^1^, which uses 43 single copy phylogenetic marker genes to assess bin completeness (Parks et al., 2015). The MSA obtained from the CheckM output was then used to generate a tree using FastTree2 (v2.1.9) (Price et al., 2010). Using the phylogenetic tree along with CheckM stats, bins were manually selected based on phylogenic classification, completeness, and contamination. In most instances the phylogenetic tree nodes were grouped in sets of three, representative of each assembly method (i.e., IDBA-ID, MEGAHIT, and metaSPAdes), which all contained the same marker lineage designation, number of genomes, number of markers, and number of marker sets. The representative bin was then chosen based on highest completeness and lowest contamination. These finalized metagenome assembled genomes (MAGs) were combined with previously obtained MAGs associated with dominant Ney Springs taxa (Trutschel et al., 2022) to investigate the core microbial community members. All assemblies were annotated or re-annotated using the KEGG GhostKoala (v2.2.) online interface (Kanehisa et al., 2016). Metabolic pathway completeness was assessed using the KeggDecoder package (Graham et al., 2018) and via manual search of KO terms for genes of interest not included in the KeggDecoder package. Metagenome data is available under the NCBI BioProject accession number PRJNA739719.

## Results and discussion

### 93 ASVs constitute Ney Springs’ cistern resident community

**Figure 1:**
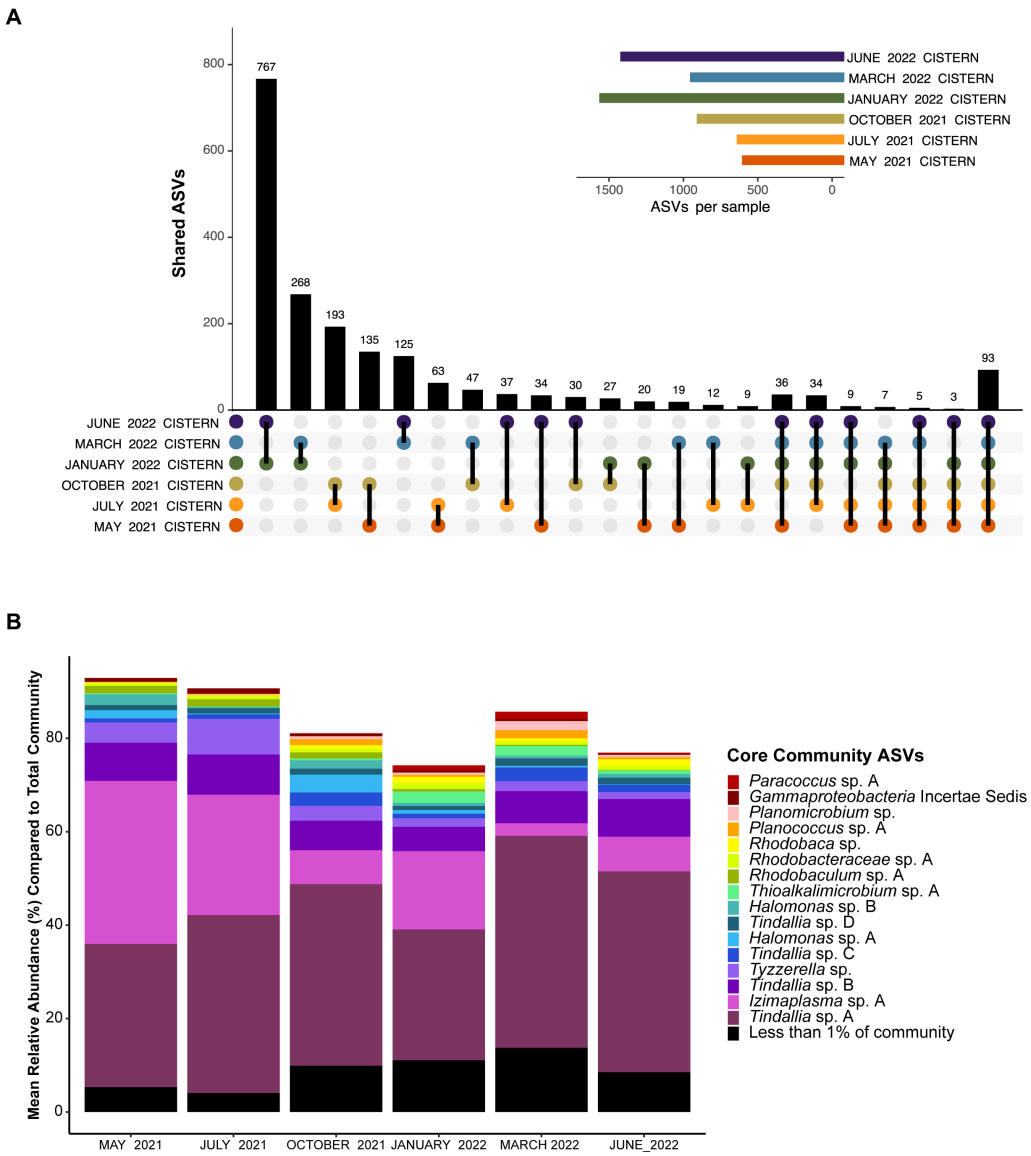
**(A)** UpSet plot showing how many unique ASVs overlap between pooled monthly samples. Plot shows overlap between all six months, at least five of the six months, and then ASVs which are only found in two of the six months sampled. The 93 ASVs found in all six months sampled represent the resident community members. The sample size for each month was *n* ≥ 3. **(B)** Barplot showing the mean relative abundance of the 93 resident community ASVs compared to the total community. Only the top 16 with a mean relative abundance of >1% are shown, which represent the core community members. The remaining 77 resident community members are grouped together. A complete list of the resident community members can be found in Supplementary Data 1.

Analysis of the pooled monthly seasonal samples revealed many transient ASVs found during only one of the sampling events, with approximately 17,000 out of the almost 20,000 ASVs detected falling into this category. These transient microorganisms are suspected to mostly come from input of debris from the surrounding environment (e.g., plants, insects, dust) into the cistern. The transient community is higher in diversity but much lower in abundance compared to the resident community, which was comprised of only 93 ASVs observed every sampling period. These 93 ASVs are referred to as the resident microbial members due to their persistent detection in the spring (Figure 1A). Notably, the resident community members were all bacteria, with Archaea only detected in low amounts in both the 16S RNA gene survey and metagenomic data for the cistern (Supplementary Datas 1, 2). This aligns with previous findings from Ney Springs which showed very little Archaeal presence (Trutschel et al., 2022). Of these resident community ASVs, the *Tindallia* and *Izimaplasma* genera consistently dominated the microbial community; seven *Tindallia* ASVs comprised 36–55% of the community and two *Izimaplasma* ASVs ranged from 3 to 36%. 16 of the resident community ASVs had an average annual abundance of greater than 1%. These 16 were deemed the core community ASVs and collectively comprised 63–87% of the community alone, while the 93 resident ASVs comprised 74–93% of the microbial community (Figure 1B).

The core community taxa found are from genera predominantly associated with alkaline environments, with many representatives previously detected in soda lakes. For example, the predominance of *Tindallia* and *Izimaplasma* species is distinct compared with other serpentinizing systems, though these taxa have been detected within soda lakes (Kevbrin et al., 1998; Vavourakis et al., 2018). Other predominant core community taxa are those belonging to the *Halomonas* and *Rhodobacteraceae* groups. Isolates from these groups have been cultured from multiple alkaline soda lake environments and have been shown to be heterotrophic sulfur oxidizers (Sorokin et al., 2000; Sorokin, 2003; Bryantseva et al., 2015; Kopejtka et al., 2017). Approximately 15% of the resident community ASVs belong to the *Rhodobacteraceae* and include the intermingled and poorly phylogenetically resolved *Paracoccus*, *Rhodobaca*, *Rhodobaculum*, *Roseibaca*, and *Roseinatronobacter* genera. The closest relative of the Gammaproteobacteria incertae sedis ASV is *Wenzhouxiangella*, another genus originally isolated from an alkaline soda lake (Sorokin et al., 2020). *Thioalkalimicrobium* (aka *Thiomicrospira*) is the only core community member also observed in high abundance in other serpentinizing system microbial communities-the Lost City and Prony Bay hydrothermal fields (Brazelton et al., 2012; Postec et al., 2015), though species have also been isolated from soda lakes as well (Sorokin et al., 2002). Ney Springs is located <640 km from Mono lake, a soda lake which shares many similar microbial members to those found in Ney Springs, such as *Halomonas, Thiomicrospira*, and *Roseinatronobacter* (Humayoun et al., 2003; Trutschel et al., 2022). The remaining core community taxa include *Planomicrobium* species, which are not known to be associated with alkaline environments, but the closely related *Planococcus* have been isolated from alkaline soils (Wang et al., 2015). There is also *Tyzzerella*, which is commonly found in the human gut microbiome, though the closest matches with our 16S rRNA sequence are from uncultured members detected in termite guts—which, are known for highly alkaline conditions that aid in digestion of plant material (Schmitt-Wagner et al., 2003). Overall, the core community taxa identified show precedence for being alkaliphiles, though this is the first time many have been detected in abundance within a serpentinizing system.

### Seasonal evaporation occurs in the Ney Springs cistern

**Figure 2:**
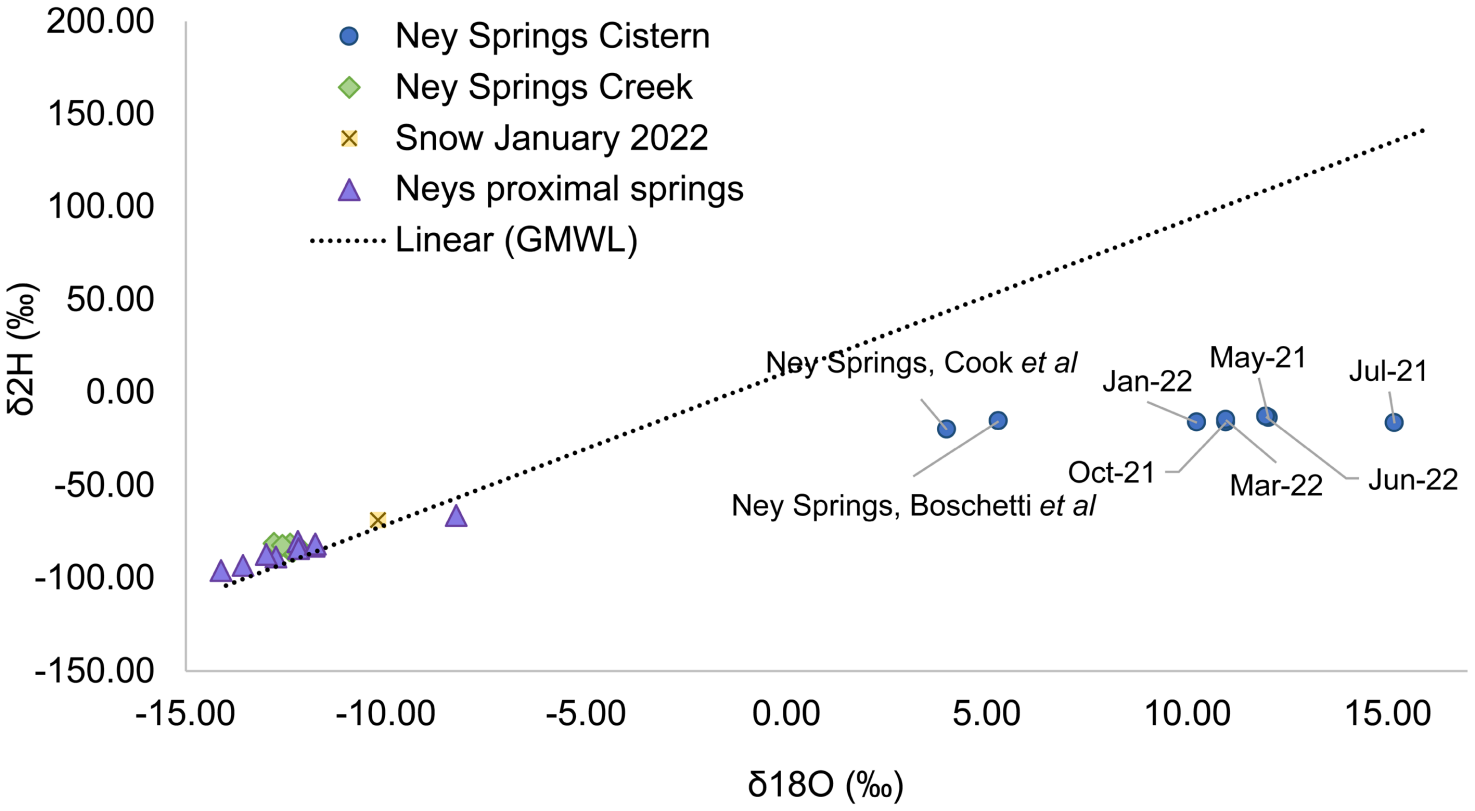
Water isotope plot showing Ney Springs cistern samples collected seasonally compared to surface water proximal springs, Ney Springs Creek, snow melt, and the global meteoric water line. Samples are differentiated by color and shape.

**Figure 3:**
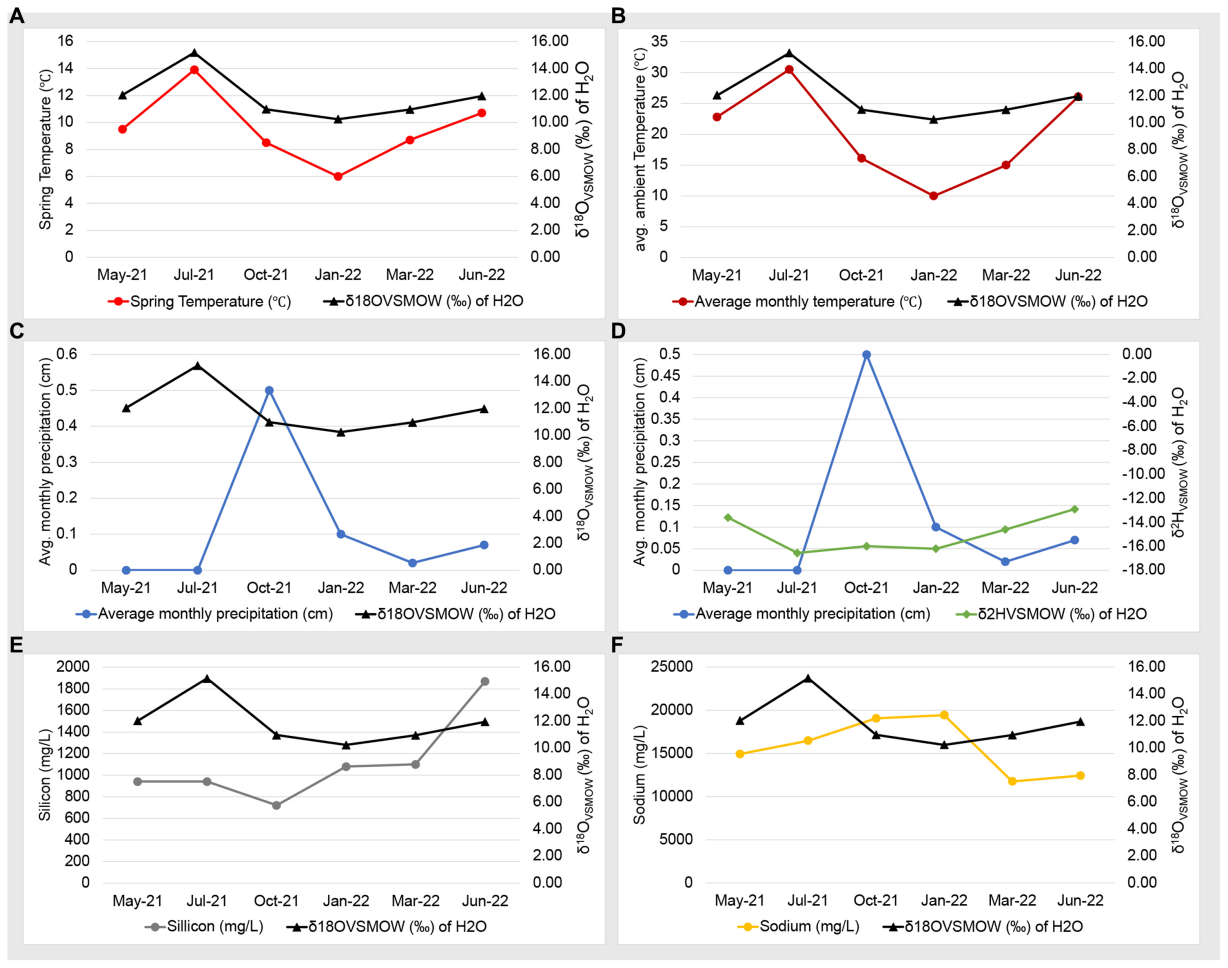
Geochemistry and water isotope time series plots. Plots consists of two *Y* axes, with data points listed in chronological order on the *X* axis. Panels are as follows: **(A)** Ney Springs cistern fluid temperature compared to δ^18^O (‰ VSMOW) of H_2_O. **(B)** Average ambient monthly temperature for greater Ney Springs area compared to δ^18^O (‰ VSMOW) of H_2_O. **(C)** Average monthly precipitation for greater Ney Springs area compared to δ^18^O (‰ VSMOW) of H_2_O. **(D)** Average monthly precipitation for greater Ney Springs area compared to δ^2^H (‰ VSMOW) of H_2_O. **(E)** Silicon concentration in Ney Springs cistern (mg/L) compared to δ^2^H (‰ VSMOW) of H_2_O. **(F)** Sodium concentration in Ney Springs cistern (mg/L) compared to δ^2^H (‰ VSMOW) of H_2_O.

Our previous work used water isotopes to demonstrate that fluids from the Ney Springs primary cistern are distinct from other water sources in the Mt. Shasta/Dunsmuir, CA area as they diverge greatly from the meteoric water line (Trutschel et al., 2022). Our seasonal analysis has now identified fluctuations within the water isotope signatures, specifically within the δ^18^O (‰ VSMOW) isotopes of H_2_O (Figure 2). This change in oxygen isotope enrichment is likely due to evaporation as the highest δ^18^O (‰ VSMOW) values are observed in July 2021 and June 2022, corresponding to the highest site temperatures, and the lowest δ^18^O (‰ VSMOW) concentrations coinciding with the lowest temperature in January 2022 (Figures 2, 3A,B). The temperature extremes for the cistern were observed in January 2022 at 6°C (external daytime temperature −0.5 to 11.7°C) and in July 2021 at 13.9°C (external daytime temperature 13.3 to 32.8°C). A strong positive correlation is seen between δ^18^O (‰ VSMOW) values and cistern temperature (correlation coefficient of 0.96) as well as δ^18^O (‰ VSMOW) values when plotted alongside average monthly temperature for the region (correlation coefficient of 0.89) (Figure 3). When comparing average monthly precipitation to changes in water isotopes, we do not see a strong correlation. Very little precipitation is observed in the region, and a decrease in neither δ^2^H (‰ VSMOW) nor δ^18^O (‰ VSMOW) was observed in Ney water isotopes during October when precipitation was greatest (Figure 3). The cistern itself has a recharge rate of 3.88 L/h and its ability to refill quickly does not appear to be influenced by meteoric input. While evaporation appears to be the main driver of seasonal changes in water isotopic signatures, evaporation and precipitation do not appear to influence concentration in redox stable geochemical species such as silicon and sodium which may be more indicative of water rock-interactions (Figures 3E,F). Sodium levels at Ney Springs are elevated compared to typical marine geochemistry, making it a likely byproduct of subsurface water-rock interactions (Feth et al., 1961). At this point Ney Springs hydrogeology and specifically how this particular spring is isolated from meteoric water remains unknown. This in addition to the variation in geochemistry that may relate to active vs. mineralized serpentinized fluids is in question, but at present there is no evidence for fluid mixing in the Ney Springs cistern.

**Figure 4:**
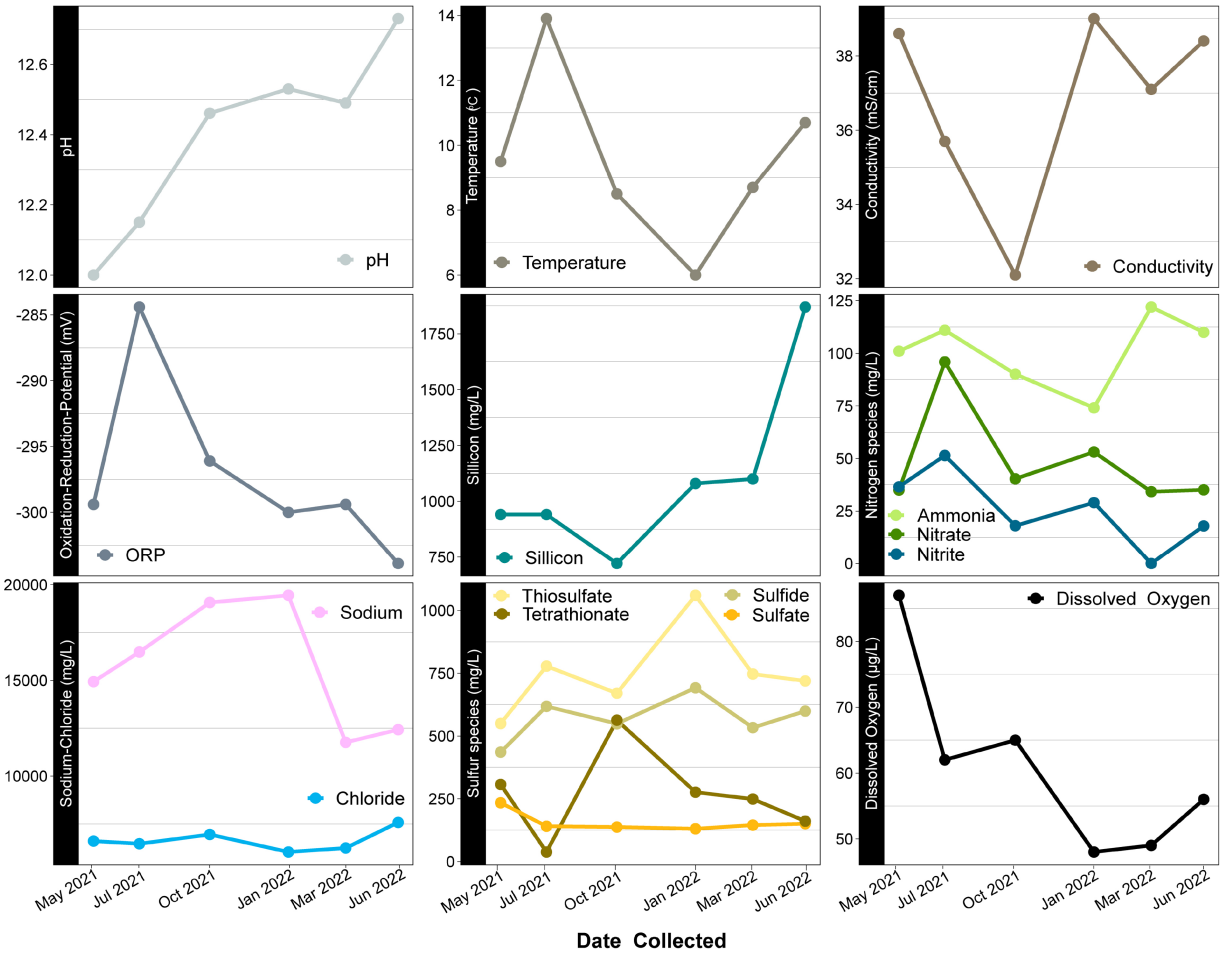
Time series scatter plots of geochemical constituents measured over a year at Ney Springs. *X* axis represents time sampled while *Y* axis specifies units for each constituent. Similar species likely to have relationships are grouped together.

Temporal variation is also observed in several redox active geochemical constituents, such as sulfur and nitrogen species. These species are more liable to be altered by microbial processes, and their variation may suggest that microbial community dynamics are driving changes that may or may not be related to other environmental parameters that change seasonally (i.e., temperature). In this system, sulfate is predicted to come from the connate nature of the deeper ground waters being influenced by the marine Franciscan subduction complex. It has previously been speculated that the sulfide present in the spring is potentially a product of microbial sulfate reduction, as it is not volcanic in nature (Feth et al., 1961). However, sulfur oxidation, which was previously shown to be a viable metabolism in this system, could also impact sulfate/sulfide concentrations (Trutschel et al., 2022). Interestingly, the balance of sulfur species changes over the course of our year sampling period. The abundance of sulfide vs. oxidized products supports the influence of microbial activity (Figure 4). While this change may be occurring at the surface level, deeper subsurface microbial activity and/or water-rock interactions could be influencing the sulfur species composition as well.

The high ammonia concentration (74–122 mg/L) in this system has been anomalous, especially compared to other characterized serpentinizing systems (Trutschel et al., 2022). It has been hypothesized that the high ammonia in Ney Springs may originate from decaying organic matter, though it is currently unclear if ancient or modern material could be the source (Waring, 1915; Feth et al., 1961). Ammonia concentrations vary over the sampling period, as do other detected nitrogen species. Nitrate (34–95 mg/L) and nitrite (0.01–51 mg/L) are also much higher than what is seen in other serpentinizing systems (Cardace et al., 2015; Crespo-Medina et al., 2017; Cook et al., 2021). The high concentration of nitrogen species within Ney Springs could come from interactions with the Franciscan Subduction Complex, but as the values are much higher than other serpentinizing systems within the same host geology (Morrill et al., 2013), this suggests the presence of additional nitrogen sources as well. Temporal variation in input from ancient marine sediment rich in organic matter could be contributing to nitrogen concentrations, as could subsurface microbial dissimilatory nitrate reduction to ammonia. However, it is also worth noting that within our system, we see particularly elevated amounts of nitrogen species during May and July of 2021, which may be due to seasonal changes in proximal environmental factors such as vegetation.

### Changes in geochemistry and abundance of core microbial community members help explain seasonal variation

**Figure 5:**
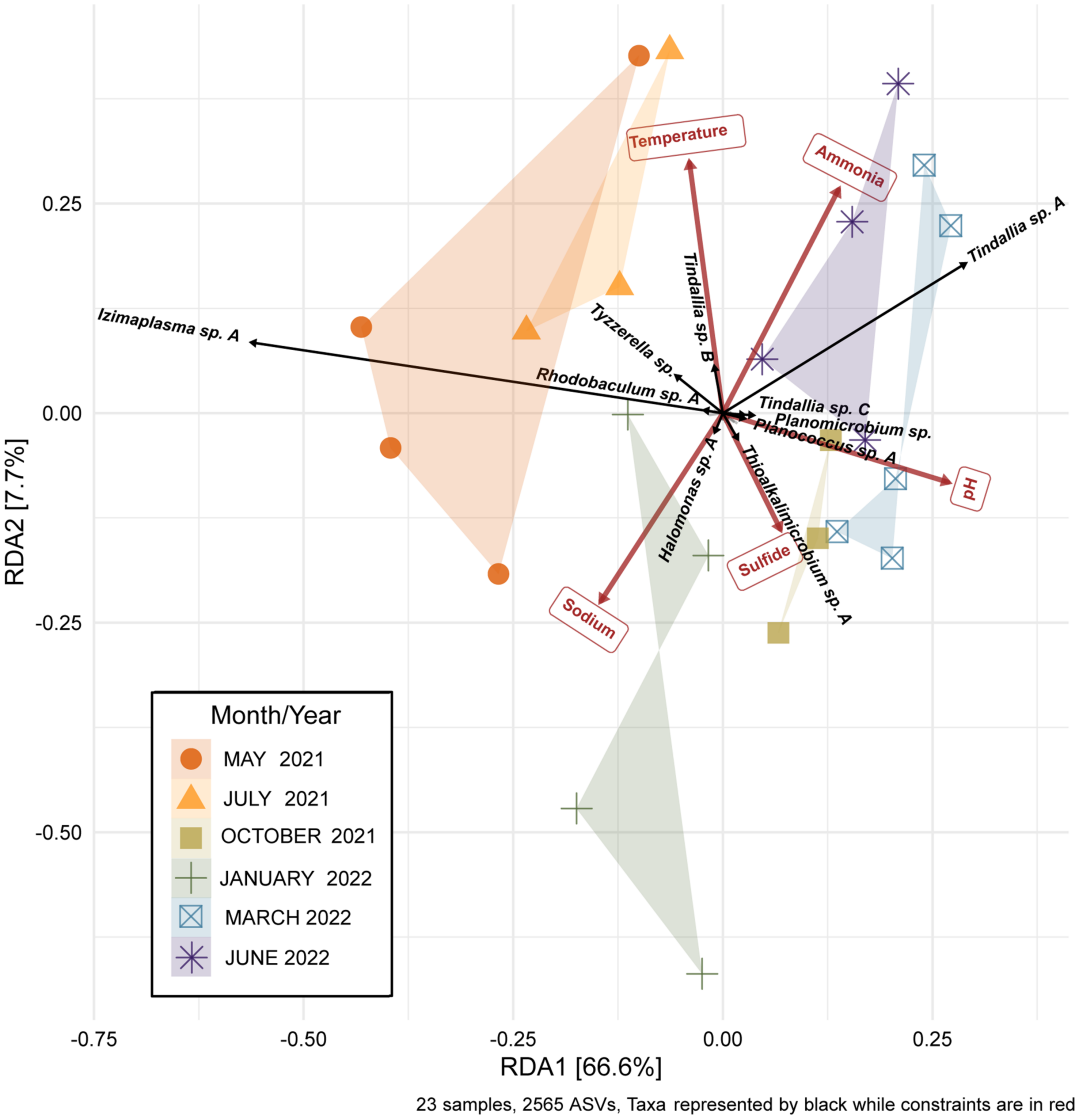
Redundancy analysis (RDA) plot of the Ney Springs cistern microbial community samples. 23 microbial community samples were collected during six different sampling events. Samples are devoid of transient ASVs, i.e., ASVs that were only encountered during one sampling event, in order to best represent the resident microbial community. Count data is transformed to be in terms of relative abundance per sample. Constrained elements were chosen based on their ability to explain variation within the microbial community and lack of overlap with one another.

While the overall microbial community composition of Ney Springs changes seasonally, all of the samples collected across the six sampling events have a similar degree of variance. Permanova/adonis results on Bray-Curtis distances calculated for the monthly samples revealed there is a significant difference in the centroids of monthly samples (pr (>F) = 0.001), and they maintain a similar homogeneity of dispersion between them and are not significantly different in dispersal pattern (betadisper, pr (>F) = 0.224). Interestingly, community structure does not appear to be solely a function of season. For example, not all summer months cluster similarly. While May 2021 and July 2021 samples cluster, the June 2022 community samples cluster near March 2022 (Figure 5). Structure is also not simply a product of linear divergence over time due to the placement of the January 2022 and October 2021 samples in between these clusters. However, a longer sampling period would be needed to determine if the community follows any sort of cyclical or oscillating pattern.

**Figure 6:**
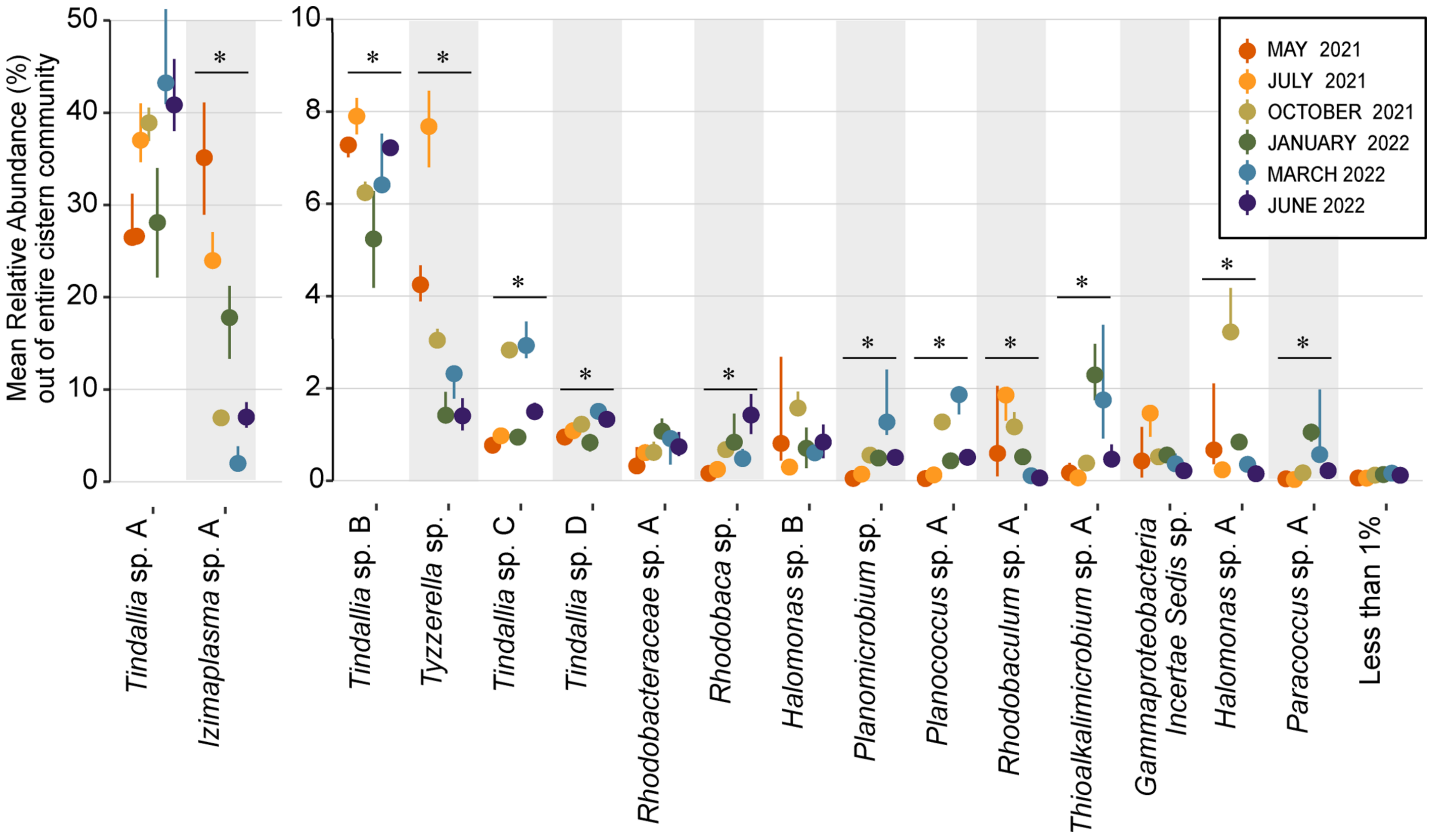
Dot plot showing change in mean relative abundance of sixteen core community ASVs that regularly comprise 1% or more of the total community. The remaining 77 resident community members are grouped together as the “Less than 1%” category. ASVs are organized by decreasing overall mean relative abundance, with groups split between two *Y* axes in order to better visualize changes in less abundant ASVs. The dots plotted represent the mean relative abundance of *n* ≥ 3 samples each month, while lines emitting from the dots represent the 95% confidence interval. ASVs that had a significant change in relative abundance between months (Kruskal-Wallis test, *p* value <0.05) are denoted with a bar and “*” above them.

The strongest correlations between taxa with particular sampling periods were seen in core community ASVs that experienced a significant increase in relative abundance within that sampling period (Figures 5, 6). Out of the 16 core community members, 12 underwent significant changes to their mean relative abundance seasonally (Kruskal-Wallis test, *p* value <0.05) (Figure 6). The greatest change in average relative abundance was observed in *Izimaplasma* sp. A, between May 2021 and March 2022 at 35% vs. 2.7% of the total microbial community, respectively, (Dunn test, p.adj. value = 0.0001). Previously, *Izimaplasma* had been observed as the most abundant microbial community member during the first sampling of Ney Springs in late May of 2019, reinforcing its observed strong association with the early summer month (Trutschel et al., 2022). Two other ASVs followed the inverse of this pattern, with their highest abundance and strongest correlation associated with March 2022 and their lowest abundance observed in May 2021. This included *Planococcus* sp. A (March 1.7% vs. May 0.05%) and the *Planomicrobium* sp. (March 1.91% vs. May 0.05%) (Dunn test, p.adj. values <0.01). Other ASVs experiencing a period of upsurge where their average relative abundance was significantly higher (Dunn test, p.adj. value<0.04) compared to two or more of the other sampling times included the *Tyzzerella* sp. during July 2021 with a maximum observed relative abundance of 7.6%, *Halomonas* sp. A in October 2021 at 3.84%, *Thioalkalimicrobium* sp. A in January 2022 at 2.41%, and *Tindallia* sp. C in March at 2.93%.

In addition to determining sampling periods’ associations with specific ASVs, a redundancy analysis (RDA) was performed to determine how much seasonal variation within the microbial community may be explained by changes in geochemistry. Constrained elements were chosen based on their seasonal variation, potential for interaction with microbial metabolism, and on their unrelatedness to one another. These parameters included pH, temperature, sodium, ammonia, and sulfide. The five independent constrained variables explained 74.3% of the variation seen between the microbial community samples (Figure 5). This revealed several potential relationships between core community taxa and constrained elements associated with metabolism, such as *Tindallia* spp. with ammonia as well as *Thioalkalimicrobium* sp. A, and *Halomonas* sp. A with sulfide. Meanwhile, changes in pH, sodium, and temperature may cause a shift in favorable growth conditions for multiple core community members in a way that broadly alters structure. This could also explain why certain sampling periods are associated more closely with these parameters (e.g., sodium with January 2022).

### Core community associated MAGs show adaptations to salinity and alkalinity

To understand the drivers of the observed correlations between species abundance, seasonality, and geochemistry, metagenome assembled genomes (MAGs) of the core microbial community were analyzed. In addition to seven previously obtained MAGs (Trutschel et al., 2022), we report nine additional MAGs used to investigate the metabolic potential of the core community members (Table 1). Three MAGs were below 95% complete (*Planococcus* bin 006, *Lachnospirales* bin 026, and *Roseinatronobacter* bin 022) and though all were present in the core microbial community, they have been omitted from further analysis due to the inability to confidently assess metabolism. *Tindallia* bin 004 was included despite its higher potential for contamination (9.27%) because it contained a 16S rRNA gene sequence that directly matched the most abundant ASV (*Tindallia* sp. A) and because all genes of interest matched *Tindallia* bin 001, which only contained 3% contamination. When assessing mechanisms for dealing with the stress of this environment, focus was placed on the organisms’ genetic potential for tolerating salinity and alkalinity. While temperature is potentially a driving feature of seasonal variation of the spring community, genome level adaptations to temperature were not investigated as the cistern temperature remained in the low mesophilic to psychrophilic range all year (6–13.9°C), and as such, we would not expect a strong genome level signature for temperature.

**Table 1:** Summary of MAGs relating to core microbial community.

MAG name	Phylum	Class	Order	Family	Genus	Completeness	Contamination	Contigs	Potential core community ASV match based on taxonomic classification	Source
March 2022-Tindalliaceae.bin004	Firmicutes_A	Clostridia	Peptostreptococcales	Tindalliaceae	JAABSW01	95.8	9.27	143	Direct match to *Tindallia* sp. A 16S rRNA	This work
May 2019-Tindalliaceae.bin001	Firmicutes_A	Clostridia	Peptostreptococcales	Tindalliaceae	–	97	3	275	*Tindallia* sp. B,C,D,	Trutschel et al. (2022)
January 2022-Izemoplasmataceae.bin006	Firmicutes	Bacilli	Izemoplasmatales	Izemoplasmataceae	CSBR16-87	98.67	0	75	*Izimaplasma* sp. A	This work
May 2019-Tenericutes.bin	Firmicutes	Bacilli	Izemoplasmatales	–	–	99	0	97	*Izimaplasma* sp. A	Trutschel et al. (2022)
^1^May 2019-Lachnospirales.bin026	Firmicutes_A	Clostridia	Lachnospirales	UBA5962	–	90	0	35	*Tyzzerella* sp.	Trutschel et al. (2022)
July 2021-Rhodobacteraceae.bin004	Proteobacteria	Alphaproteobacteria	Rhodobacterales	Rhodobacteraceae	Tabrizicola	95.52	5.19	330	*Paracoccus* sp. A	This work
July 2021-Rhodobacteraceae.bin017	Proteobacteria	Alphaproteobacteria	Rhodobacterales	Rhodobacteraceae	Yoonia	98.99	0.84	329	*Rhodobaculum* sp. A	This work
July 2021-Rhodobacteraceae.bin021	Proteobacteria	Alphaproteobacteria	Rhodobacterales	Rhodobacteraceae	Roseibaca	98.94	0.61	330	*Rhodobaculum* sp. A	This work
May 2019-Rhodobacteracea.bin019	Proteobacteria	Alphaproteobacteria	Rhodobacterales	Rhodobacteraceae	–	96	1.1	168	*Rhodobacteraceae* sp. A	Trutschel et al. (2022)
^1^March 2022-Roseinatronobacter.bin022	Proteobacteria	Alphaproteobacteria	Rhodobacterales	Rhodobacteraceae	Roseinatronobacter	74.14	8.75	58	*Rhodobacteraceae* sp. A	This work
May 2019-Halomonas.bin014	Proteobacteria	Gammaproteobacteria	Pseudomonadales	Halomonadaceae	Halomonas	99	6.1	229	*Halomonas* sp. A,B	Trutschel et al. (2022)
^1^March 2022-Planococcus.bin006	Firmicutes	Bacilli	Bacillales	Planococcaceae	Planococcus	54.52	4.52	275	*Planococcus sp*. A, *Planomicrobium* sp.	This work
^2^July 2021-Thiomicrospira.bin012	Proteobacteria	Gammaproteobacteria	Thiomicrospirales	Thiomicrospiraceae	Thiomicrospira	99.39	0	164	*Thioalkalimicrobium* sp. A	This work
March 2022-Wenzhouxiangella.bin008	Proteobacteria	Gammaproteobacteria	Xanthomonadales	Wenzhouxiangellaceae	Wenzhouxiangella	98.71	2.04	276	*Gammaproteobacteria Incertae Sedis* sp.	This work
May 2019-Wenzhouxiangella.bin008	Proteobacteria	Gammaproteobacteria	Xanthomonadales	Wenzhouxiangellaceae	Wenzhouxiangella	96	1.2	56	*Gammaproteobacteria Incertae Sedis* sp.	Trutschel et al. (2022)
May 2019-Wenzhouxiangella.bin027	Proteobacteria	Gammaproteobacteria	Xanthomonadales	Wenzhouxiangellaceae	Wenzhouxiangella	98	4.6	71	*Gammaproteobacteria Incertae Sedis* sp.	Trutschel et al. (2022)

1 These MAGs omitted from metabolic discussion in paper due to less than 95% completeness.

2 At the genus level this organism may be classified as Thiomicrospira or Thioalkalimicrobium.

**Figure 7:**
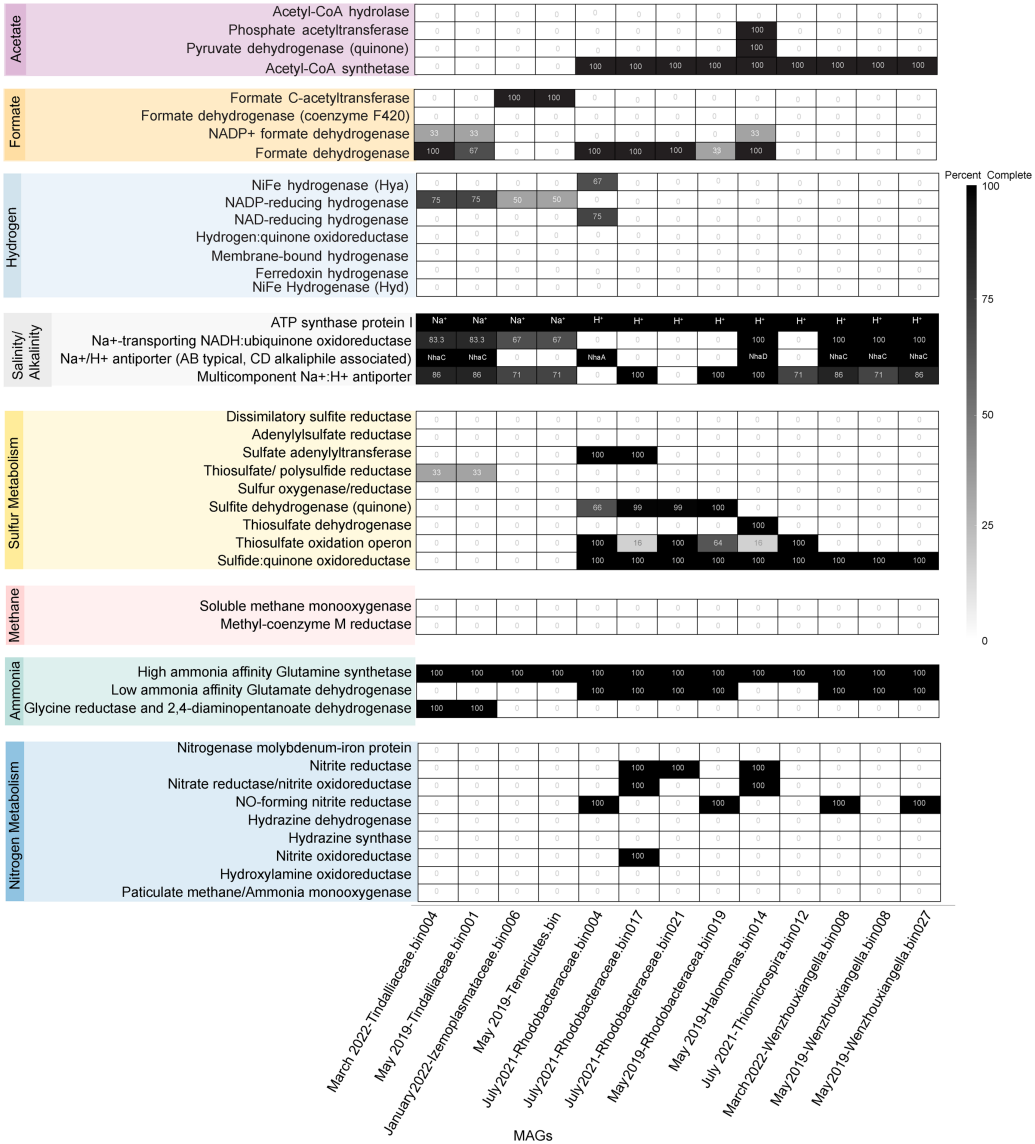
Heatmap of core community associated MAGs with selected marker proteins relating to nitrogen, sulfur, methane, hydrogen, formate, and acetate metabolism or are associated with alkalinity and salinity tolerance. Only MAGs that had greater than 95% completeness are shown within the Heatmap. The black boxes for ATP synthase protein I have all necessary subunits for an F-type ATPase (AtpFBCHGDAE) and instead specify whether an organism is likely to encode for a Na^+^ or H^+^ binding ATP synthase based on amino acid sequence. For organisms that do contain the gene homolog, the black boxes for the Na^+^/H^+^ antiporter specify if the organism is likely to contain NhaA, C or D, with Nha C and D originally characterized in and often associated with alkaliphiles.

**Figure 8:**
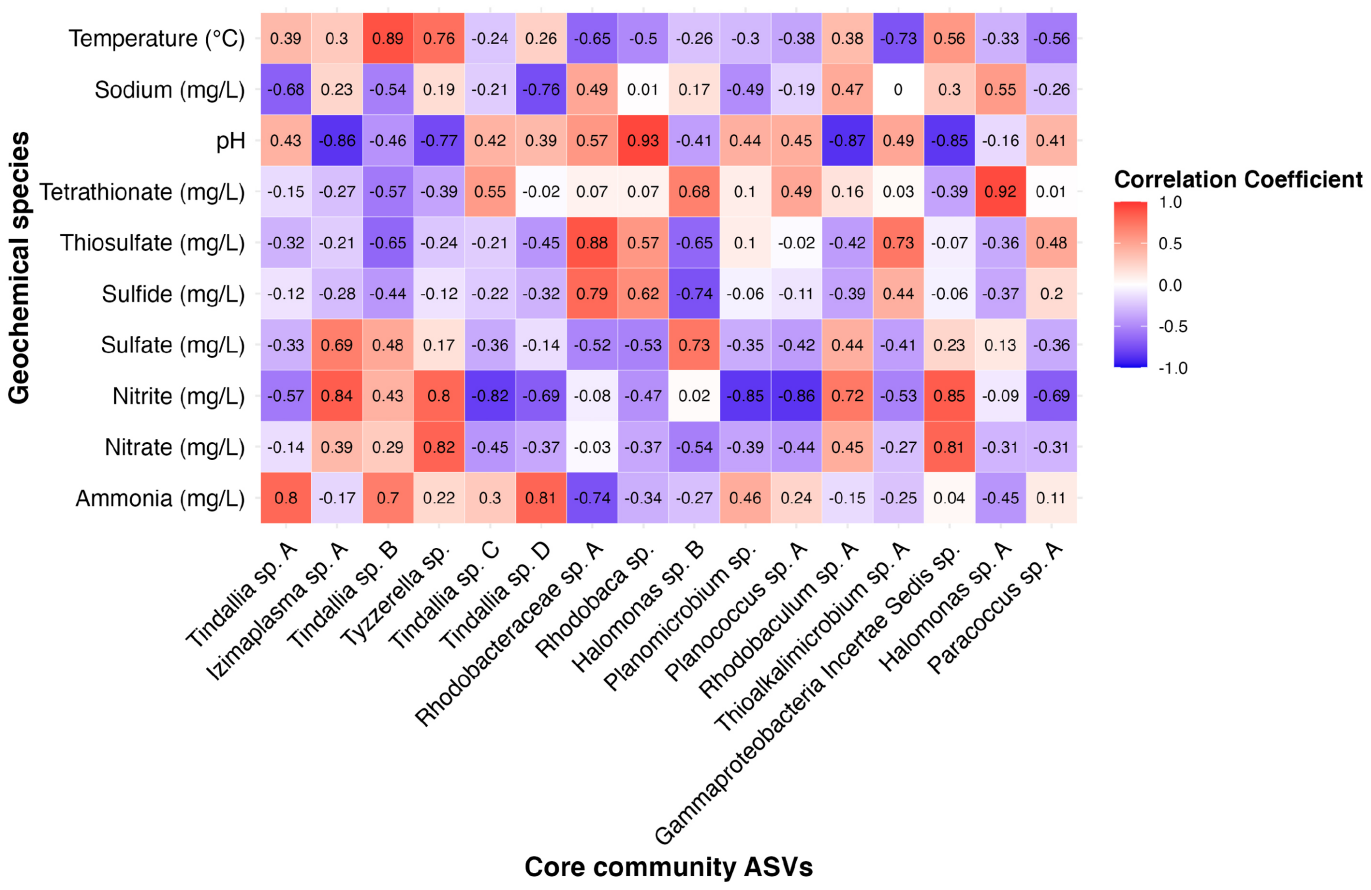
Heatmap showing correlation coefficient values between the relative abundance of 16 core community ASVs with geochemical constituents of interest as determined by results of RDA analysis.

**Figure 9:**
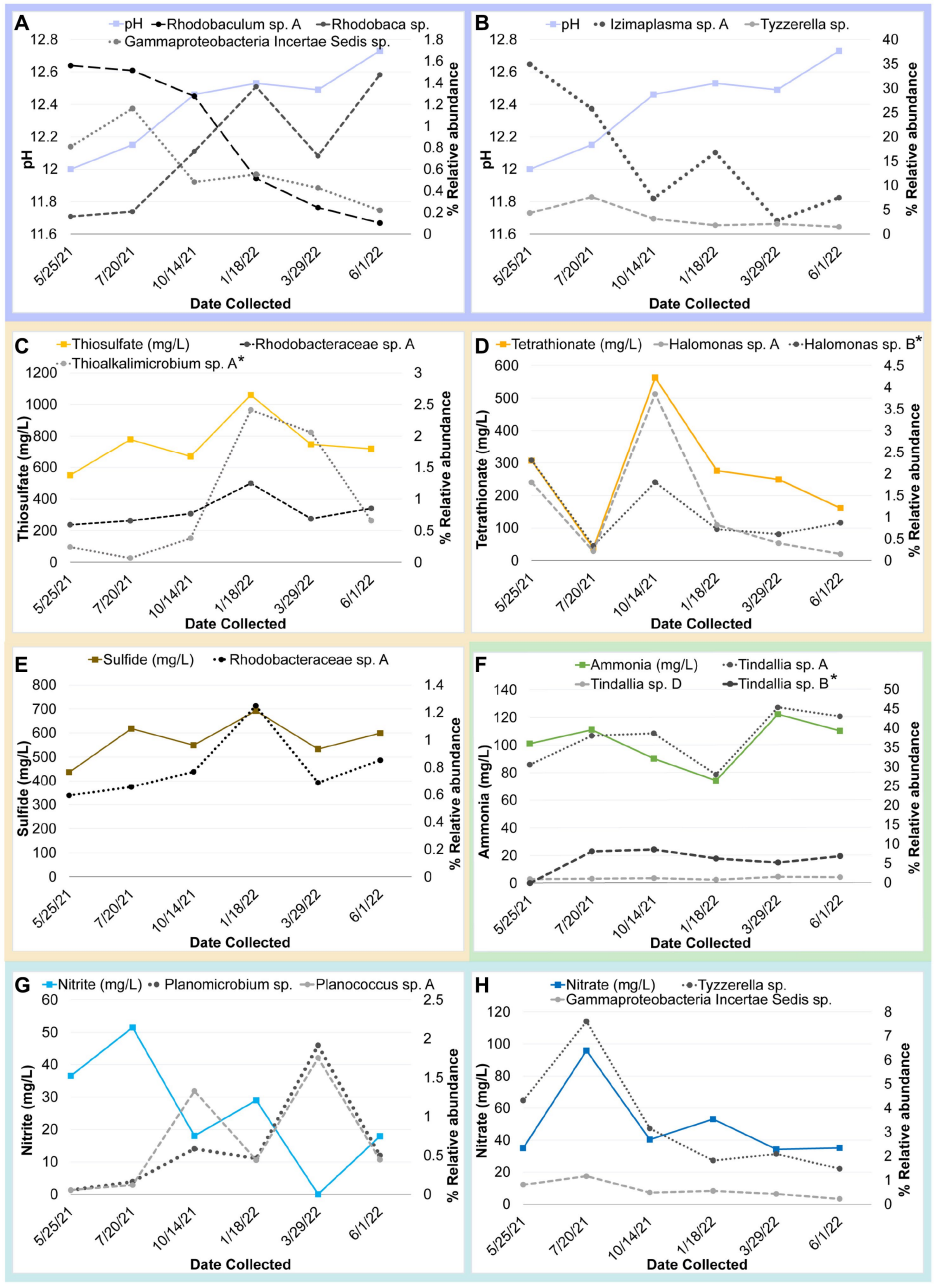
Timeseries plot of changes in core community ASV relative abundance that may be related seasonal changes in a geochemical constituent as identified by a correlation coefficient value at or above 0.80 unless otherwise noted (*). Each plot shows the relative abundance of one or more core community ASVs plotted alongside a different variable. Panels are as follows with correlation coefficient values for each ASV indicated in parentheses: **(A)** Change in pH compared to relative abundance of Gammaproteobacteria incertae Sedis sp. (−0.85), *Rhodobaculum* sp. (−0.87), and *Rhodobaca* sp. (0.93). **(B)** Change in pH compared to relative abundance of *Izimaplasma* sp. A (−0.86) and *Tyzzerella* sp. (−0.77) **(C)** Change in thiosulfate compared to relative abundance of *Thioalkalimicrobium/Thiomicrospira* sp. A (0.73*) and *Rhodobacteraceae* sp. A (0.88). **(D)** Change in tetrathionate compared to relative abundance of *Halomonas* sp. A and B (0.92 and 0.68*). **(E)** Change in sulfide compared to relative abundance of *Rhodobacteraceae* sp. A (0.79). **(F)** Change in ammonia compared to relative abundance of *Tindallia* sp. A, B and D (0.8, 0.7*, 0.81, respectively). **(G)** Change in nitrite compared to relative abundance of *Planococcus* sp. A (−0.86) and *Planomicrobium* sp. (−0.85). **(H)** Change in nitrate compared to relative abundance of *Tyzzerella* sp. (0.82) and Gammaproteobacteria incertae Sedis sp. (0.81).

Many of the MAGs encoded genes associated with salinity and alkalinity tolerance such as Na^+^/H^+^ antiporters Mrp and/or Nha (Figure 7). Mrp antiporters are often essential for maintaining an electrochemical gradient in alkaline and marine conditions by pumping sodium ions out while pumping protons in (Ito et al., 2017). Homologs of the Na^+^/H^+^ antiporter NhaD found in the *Halomonas* MAG do not exhibit activity below pH 8 and have thus far only been found in alkaliphiles (Nozaki et al., 1998). NhaC homologs, which are detected in the *Tindallia* and *Wenzhouxiangella* MAGs, have been shown to be necessary for growth in alkaliphilic conditions for several *Bacillus* sp. (Ito et al., 1997; Krulwich et al., 1997). Putative sodium pumping NADH-coQ reductase (Nqr) was also observed in many of the MAGs, which can help maintain the electrochemical gradient under alkaline conditions in conjunction with the H^+^/Na^+^ antiporters by pumping sodium out (Vorburger et al., 2016). As described previously, most of the MAGs appear to encode for H^+^ binding rather than Na^+^ binding ATPases based on amino acid sequence despite the low concentrations of H^+^ at pH 12 (Mulkidjanian et al., 2008; Trutschel et al., 2022). The exceptions to this are the *Tindalliaceae* and *Izemoplasmataceae* MAGs which are predicted to contain Na^+^ binding F-type ATPases and are notably the most abundant taxa in the system (Figure 7). *Tindallia* sp. A and D exhibit a slight negative correlation with sodium respectively, but no other core community ASVs have a suggested strong relationship with sodium (Figure 8). Notably, the relative abundance of *Izimaplasma* sp. A is negatively correlated with pH, while only the *Rhodobaca* sp. A ASV was strongly positively correlated with an increase in pH (Figures 8, 9A,B). Previously an isolate from the *Roseinatronobacter*-*Rhodobaca* cluster of the *Rhodobacteraceae* family was isolated from Ney Springs and was found capable of growth in pH 12.4 media (Trutschel et al., 2022), suggesting that some members of this clade may be better at tolerating high pH conditions.

### Metagenomic information shows potential for ammonia production by most abundant core community member

The source of ammonia within Ney Springs is unknown, but may be linked to current or past microbial activity. The potential for generation of ammonia through denitrification (DNRA) is observed in the *Rhodobacteraceae* and *Halomonas* MAGs, which each encode nitrate and nitrite reductases (NarGH/NapAB and NirBD) (Figure 7), however none of the *Rhodobacteraceae* or *Halomonas* ASVs exhibit a strong correlation coefficient with nitrite, nitrate, or ammonia (Figure 8). Only *Planococcus* sp. A and the *Planomicrobium* sp. were observed to have a relationship with nitrite (Figure 9G), while the *Tyzzerella* and Gammaproteobacteria Incertae Sedis spp. were the only ASVs positively correlated with nitrate (Figure 9H). Conversely, *Tindallia* sp. A, B and D are all positively correlated with ammonia (Figures 8, 9F). The *Tindallia* MAGs encode the enzymes necessary for Stickland reactions from glycine and ornithine (GrdABE and Ord), which have been shown to produce ammonia (Sangavai and Chellapandi, 2017). *Tindallia magadii*, the type-strain of the genus, has been observed producing upwards of 30 mM of ammonium over a 60 h period when grown in culture with 2 g/L arginine and ornithine as the initial substrate (Kevbrin et al., 1998). Stickland reaction in members of the *Peptostreptococcaceae* are cited as the most abundant ammonia producing organisms within the rumen, with several strains capable of producing up to 0.4 mM per mg of protein per minute (Paster et al., 1993; Sangavai and Chellapandi, 2017). Given the high concentrations of ammonia generally produced by these groups, it is predicted that these organisms have adaptations for ammonia tolerance, though there is little insight into what these genetic adaptations may be.

### Lack of hydrogen and methane metabolism amongst core community members

Ammonia is hypothesized to be one of key driving factors of community composition within this environment, and the likely reason we do not observe methanogens or methane oxidizers typically associated with serpentinizing systems within Ney Springs (Trutschel et al., 2022). The abundance of free molecular ammonia (NH_3_ as opposed to NH_4_^+^) potentially places strong selective pressure on microbial inhabitants due to its increased membrane passivity (Kayhanian, 1999). Both ammonia and methane associated metabolisms are known to be inhibited by high ammonia concentrations (Lehtovirta-Morley, 2018; Yan et al., 2020). No evidence of potential ammonia oxidation (AmoA or Hzo), nor methanogenesis or methane oxidation (McrA, MmoA or PmoA) was observed within the core community MAGs (Figure 7), which concurs with previous results showing a lack of evidence for these metabolisms (Cook et al., 2021; Trutschel et al., 2022). Similarly, there were very few potential hydrogenases detected within the core community MAGs (Figure 7). Partially complete NAD (HoxFUY) and NADP-reducing (HndBCD or HndCD) hydrogenases were found in five of the MAGs. A partially complete NiFe hydrogenase (HyaBC) was found within *Rhodobacteraceae* bin 004, but it was missing the small subunit (HyaA). This could suggest a loss of gene function in these organisms. Hydrogen has been measured at exceptionally low concentrations at Ney Springs when compared to other serpentinizing systems. Bubbles that arise from the bottom of the cistern have consistently contained around 0.02 atm hydrogen by volume, while dissolved hydrogen was measured at <0.01 mg/L (Mariner et al., 2003; Trutschel et al., 2022). Although thermodynamically favorable in this system, hydrogen oxidation is likely limited due to the low concentration of hydrogen available within the cistern (Trutschel et al., 2022). Acetate and formate represent other potential energy sources that may be formed via serpentinization. Many of the MAGs did encode for putative formate dehydrogenases (FdoGHI) (Figure 7), with many of the *Rhodobacteraceae* MAGs containing multiple copies. The *Izemoplasmataceae/Tenericutes* MAGs also contained formate C-acetyltransferase (PflAD). Other than the *Tindalliaceae* and *Izemoplasmataceae/Tenericutes* MAGs, the core community members all contained Acetyl-CoA synthetase (ACS). *Rhodobacteraceae* and *Halomonas* spp. have been isolated from the system previously and have been observed using acetate as a carbon/energy source (Trutschel et al., 2022).

### Temporal fluctuation in sulfur species concentrations associated with putative sulfur-oxidizing core community members

Another peculiar aspect of Ney Springs is the abundance of sulfide, which is not commonly found in terrestrial serpentinizing systems. Sulfide is found in marine serpentinizing systems such as the Lost City [2–32 mg/L (Schrenk et al., 2004)], but it is often orders of magnitude higher at Ney Springs (430–700 mg/L). Despite the abundance of sulfide and theoretical energy available for sulfate-reducing metabolic reactions, we have once again found little genetic evidence of microbial sulfide production via dissimilatory sulfate reduction or anaerobic methane oxidation using sulfate as a terminal electron acceptor (Trutschel et al., 2022). We did not detect methyl-coenzyme M reductase (McrA) or dissimilatory sulfate reductase (DsrAB) within the core microbial community associated MAGs, though a putative DsrAB was previously found in a MAG associated with resident community member *Desulfurivibrio* (Figure 7; Trutschel et al., 2022). Two *Rhodobacteraceae* MAGs putatively contain sulfate adenylyltransferase (Sat), which is likely to be involved in sulfur assimilation but has also been implicated in dissimilatory sulfur oxidation in this organism (Yu et al., 2007; Parey et al., 2013). Evidence of sulfur oxidation is much more prevalent in the core community members, as all the core community MAGs except those belonging to the *Tindalliaceae* and *Izemoplasmataceae* have the potential to engage in some form of sulfur species oxidation. The *Thiomicrospira/Thioalkalimicrobium* MAG contains Sqr (sulfide:quinone oxidoreductase) along with SoxXYZABCD (sulfur oxidation operon) and is predicted to oxidize sulfur species completely to sulfate. *Thioalkalimicrobium* sp. A relative abundance has a slight positive correlation with thiosulfate (Figure 8) and is most abundant when sulfide and thiosulfate are highest in January 2022. MAGs classified as *Rhodobacteraceae* all contain Sqr, have varying degrees of completeness of the Sox sulfur oxidation pathway, and all contain a complete or almost complete SoeABC (quinone sulfite dehydrogenase). Despite this putative evidence, only *Rhodobacteraceae* sp. A exhibits a positive correlation with sulfide and thiosulfate concentrations within the cistern (Figure 8), as its abundance is highest when thiosulfate and sulfide are also at their highest and conversely low when these concentrations are also low (Figures 9C,E). The *Halomonas* MAG only contains SoxZ (thiosulfate oxidation carrier protein), but does contain thiosulfate dehydrogenase (TsdA), an alternate thiosulfate oxidizing protein (Denkmann et al., 2012). This pathway produces tetrathionate as an end-product, which is not observed in organisms only utilizing the Sox system (Kelly et al., 1997; Grabarczyk and Berks, 2017). The changes in relative abundance of the *Halomonas* sp. A and B ASVs tracks well with changes in tetrathionate concentration within the cistern over time (Figure 9D) and *Halomonas* sp. A has a very high positive correlation coefficient with tetrathionate (Figure 8). A *Halomonas* isolate from Ney Springs has previously been shown to oxidize thiosulfate to tetrathionate *in vitro* as well, confirming this as a likely product produced by these organisms in the environment (Trutschel et al., 2022).

### Implications

Since the discovery of active serpentinization in the Coast Range ophiolite many serpentinizing systems have been identified by Barnes in Northern California, including what is now the Coast Range Ophiolite Microbial Observatory, The Cedars, and Ney Springs (Barnes et al., 1967, 1972; Barnes and O’Neil, 1969). The investigation of Ney Springs has allowed us greater insight into the ecology of terrestrial serpentinizing systems and the role host geology and microbial metabolism have on shaping geochemistry. Serpentinizing springs like Ney are commonly studied as windows into subsurface microbial communities and food webs that subsist on the reduced compounds generated by the serpentinzation reaction. Notably, these systems maintain their high pH and much of their geochemistry despite surface exposure, which results in a specialized microbial community. This can be seen within Ney Springs, with the resident community members making up the overwhelming majority of this interface microbial community. Using ASVs as the final denominator may produce an artifact of a seemingly large introduced community, but this more conservative method is preferred since it allowed us to focus on a limited number of well-established core taxa adapted to the polyextreme conditions of Ney Springs. By further identifying the putative adaptations and metabolic capabilities of these core community members, we could then assess the potential influence these organisms have on their environment and how that may explain temporal variation observed in the geochemistry.

The putative role of these core community members at Ney Springs is of interest, as they are likely driving temporal geochemical changes in the spring through their metabolisms. Within the core community, a few members had strong associations with changing geochemical parameters, and the metabolic potential we observed in their corresponding MAGs supports the capacity to use or produce these geochemical species. This was seen clearly with the *Tindallia* taxa and their correlation with ammonia concentrations. While additional experiments will be necessary to confirm that the *Tindallia* species detected are capable of excess ammonia generation, these findings represent the first plausible explanation with evidence for the profuse ammonia found within this environment. Though ammonia is a stressor, and is not common in many naturally occurring alkaline environments, it has been shown to inhibit microbial activities in bioreactors that experience ammonia buildup over time (Kayhanian, 1999; Leejeerajumnean et al., 2000). Similarly, while we have not yet observed this in other serpentinizing systems on Earth, the co-occurrence of high ammonia concentrations and serpentinization end products (e.g., hydrogen) have been detected on icy moons such as Enceladus (Vance et al., 2007; Waite et al., 2009). Understanding how ammonia impacts microbial metabolism and viability is an astrobiologically relevant question that could be further investigated at Ney Springs.

While the source of the sulfide at Ney Springs remains unclear, this work points to a metabolically diverse group of sulfur-oxidizing microbes that may use sulfide, thiosulfate, or elemental sulfur found within the spring. The complex role of sulfur intermediates within hyperalkaline environments is understudied, though many species, such as polysulfides and thiosulfate, have increased stability at high pH and are much more abundant and biologically available under these conditions (Van den Bosh et al., 2008; Findlay, 2016). Though best observed in the case of *Halomonas* and tetrathionate, other core community species may be producing and consuming these less studied sulfur intermediates. *Thiomicrospira* and members of the *Rhodobacteraceae* were more abundant when sulfide and thiosulfate were at their highest, supporting a potential link between the energy available for sulfur oxidation and these populations. Organisms like *Thiomicrospira* are obligate chemolithoautotrophs and the majority of *Rhodobacteraceae* from this environment are likely chemolithoheterotrophs. A *Rhodobacteraceae* isolate from this cistern, as well as closely related members of this family isolated from soda lakes, have been previously described as chemolithoheterotrophs and their ability to supplement energy through sulfur oxidation could explain their increased abundance during times of higher reduced sulfur species availability (Sorokin, 2003; Trutschel et al., 2022). As such, *Rhodobacteraceae* populations may be more linked to carbon pools rather than sulfur species, though at present we have only low-resolution measurements for DOC/TOC from Ney Springs and cannot identify which carbon species are present and potentially bioavailable.

Future work within Ney Springs will focus on the role of carbon speciation and how it shapes the microbial community, as many of the core community species identified did not appear to have a strong association with the geochemical constraints chosen, such as seen with *Izimaplasma*. These organisms have been twice observed having a period of significantly increased abundance within late May, but the driving factor for this bloom has yet to be identified. Potentially increased organic availability via exogenous carbon input from detritus could explain this, but further investigation is required. Other potential impacts on organism abundance are their relationships with one another. Organisms with similar metabolisms, like *Izimaplasma* and *Planocococcacae* species, which are both putative simple sugar fermenters, may face competition with one another. Similarly, a decrease in exogenous organic carbon input utilized by many of the abundant heterotrophic and/or fermentative taxa may then allow for the increased abundance of autotrophic organisms like *Thiomicrospira*. Additional work with enrichments and *in situ* activity assays may help identify which organisms are most active within this environment and are in direct competition with one another for resources. The role subsurface processes play in introducing or supporting different microbial taxa observed in this system remains to be explored. Certain geochemical parameters vary temporally with no seasonal pattern, and could be a function of differences in host rock interactions. In addition, subsurface microbial processes that are feasible but not observed in the surface community (e.g., sulfate reduction or anaerobic methane oxidation) could also be impacting the spring, though we currently lack evidence for these activities.

## Data availability statement

The datasets presented in this study can be found in online repositories. The names of the repository/repositories and accession number(s) can be found in the article/Supplementary material.

## Author contributions

LT, BK, JS, GC, and AR performed field sampling and data collection for Ney Springs. LT and BK performed geochemical analyses and interpretations. LT performed metagenomic analyses primarily with assistance from GC. LT performed statistical analyses and visualization. LT and AR are the primary authors of the manuscript with editing by BK, JS, and GC. AR and BK performed funding acquisition. All authors contributed to the article and approved the submitted version.

## Funding

Funding and salary support for LT, BK, and AR have been provided by the NSF-EAR LowTemp Geochemistry Geobiology award 2025687 and NASA-Roses Exobiology Program grant number (80NSSC21K0482). LT received a Lewis and Clark Field work in Astrobiology fellowship and the University of Cincinnati Dr. Stacy Pfaller memorial scholarship.

## Conflict of interest

The authors declare that the research was conducted in the absence of any commercial or financial relationships that could be construed as a potential conflict of interest.

## Publisher’s note

All claims expressed in this article are solely those of the authors and do not necessarily represent those of their affiliated organizations, or those of the publisher, the editors and the reviewers. Any product that may be evaluated in this article, or claim that may be made by its manufacturer, is not guaranteed or endorsed by the publisher.

## Footnotes

^1^ http://hmmer.janelia.org
