# Supplementary figures and images for: Determining resident microbial community members and their correlations with geochemistry in a serpentinizing spring
Source: Front Microbiol. 2023 Jun 15;14:1182497. doi: 10.3389/fmicb.2023.1182497 (PMC10308030; doi:10.3389/fmicb.2023.1182497)

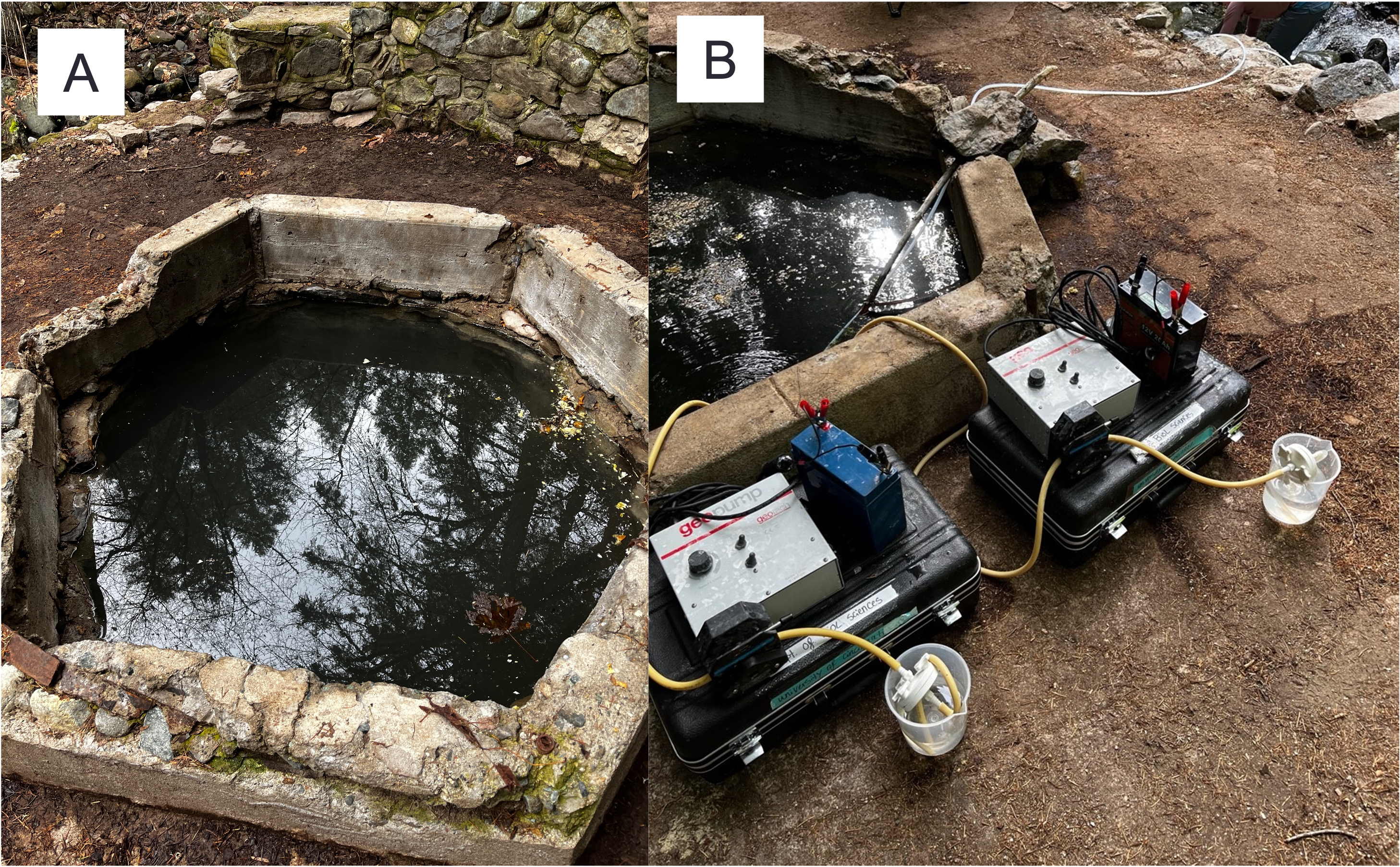

Supplement: Supplementary file 3 [file Image_1.png]
